# Une épine végétale méconnue simulant une tumeur osseuse de la main chez l’enfant

**DOI:** 10.11604/pamj.2020.35.66.15431

**Published:** 2020-03-06

**Authors:** Mohammed Tazi Charki, Moulay Abderahmane Afifi

**Affiliations:** 1 Service d’Orthopédie-Traumatologie Pédiatrique, CHU Hassan II, Université Sidi Mohammed Ben Abdellah, Fès, Maroc

**Keywords:** Epine végétale, tumeur osseuse, réaction périostée, méconnue, Plant thorn, bone tumor, periosteal reaction, unrecognized

## Image en médecine

Soft-tissue foreign bodies are common in children, particularly in the hands. They are often ignored or difficult to diagnose, especially since they are an organic foreign body causing granulomas with osteolytic lesions and/or periosteal reactions which can simulate infectious lesions or a tumor. We here report the case of a child admitted for hand tumor with periosteal reaction in whom surgical exploration found plant thorn. We here report the case of an 18-months old infant with no particular past medical history admitted to the Emergency Department due to a swelling on the back of the hand which had grown during the three weeks before its admission, without trauma. Clinical examination showed a hard, painless swelling on the back of the right hand at the level of the second metacarpal measuring 1.5 cm in diameter without inflammatory signs (Panel A). Standard radiographic evaluation of the hand revealed periosteal reaction with Codman’s triangle at the level of the outer edge of the diaphysis of the second metacarpal, with a second linear opacity next to the metaphysis (Panel B). Laboratory tests were normal. The diagnosis of bone tumor was suspected. Small incision biopsy was performed. Surgical exploration objectified a plant spine near the bone (Panel C) with detached periosteum and subperiosteal collection of shady liquid. Bone biopsy was performed. Cytobacteriologic study of the fluid was negative. Anatomo-pathological examination showed loose fibrous tissue with fibroblasts, with bone trabeculae without signs of malignancy. The infant received oral antibiotic therapy with good clinical and radiological outcome.

Les corps étrangers (CE) au sein des tissus mous sont fréquents chez les enfants, en particulier au niveau des mains. Elles sont souvent méconnues ou difficile à diagnostiquer, d’autant plus qu’il s’agit d’un CE d’origine organique, induisant des granulomes avec des lésions ostéolytiques et/ou des réactions périostées qui peuvent simuler des lésions d’origine infectieuse ou tumorale. Nous illustrons cette éventualité diagnostique par le cas d’un enfant admis pour une tumeur de main avec une réaction périostée chez qui l’exploration chirurgicale a trouvé une épine végétale. Il s’agit d’un nourrisson de 18 mois, sans antécédents pathologiques notables, admis aux urgences pour une tuméfaction du dos de la main évoluant depuis trois semaines avant son admission, sans notion de traumatisme. L’examen clinique avait mis en évidence une tuméfaction du dos de la main droite en regard du deuxième métacarpien faisant 1,5 cm de diamètre, dure, indolore, sans signes inflammatoires en regard (Panel A). La radiographie standard de la main a montré une réaction périostée type éperon de Codmann en regard du bord externe de la diaphyse du deuxième métacarpien, avec une deuxième opacité linéaire en regard de la métaphyse (Panel B). Le bilan biologique était normal. Le diagnostic d’une tumeur osseuse a été suspecté. Une incision limitée pour biopsie a été réalisée. L’exploration a objectivé une épine végétale juxta-osseuse (Panel C) avec périoste décollé et une collection sous-périostée faite d’un liquide louche. Une biopsie osseuse a été réalisée. L’étude cytobactériologique du liquide était négative. L’examen anatomopathologique avait trouvé un tissu fibreux lâche comportant des fibroblastes, avec des travées osseuses sans signes de malignité. L’enfant a été mis sous antibiothérapie par voie orale, avec bonne évolution clinique et radiologique.

**Figure 1:**
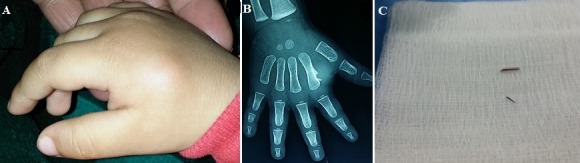
(A) Aspect clinique de la tuméfaction; (B) radiographie de la main montrant la réaction périostée; (C) épines végétales juxta-osseuses extraites

